# *APOE* ε4 linked effects on clinical features and neuropathology in dementia with Lewy bodies

**DOI:** 10.1002/alz.70795

**Published:** 2025-10-11

**Authors:** Rong Ye, Anna E. Goodheart, Joseph J. Locascio, Erin C. Peterec, Patrick Stancu, Yanhong Wang, Stephen N. Gomperts

**Affiliations:** ^1^ Department of Neurology, Massachusetts General Hospital Boston Massachusetts USA; ^2^ Harvard Medical School Boston Massachusetts USA

**Keywords:** apolipoprotein E, dementia with Lewy bodies, fluctuating cognition, neuropathology, visual hallucination

## Abstract

**INTRODUCTION:**

Apolipoprotein E (*APOE*) ε4 is a strong genetic risk factor for Alzheimer's disease (AD) neuropathologic changes, but its contribution to clinical features and pathology in dementia with Lewy bodies (DLB) is less clear.

**METHODS:**

Using the National Alzheimer Coordinating Center dataset, we investigated *APOE* ε4‐associated effects on DLB core clinical features and neuropathology.

**RESULTS:**

In analyses of *APOE* ε4 carriers, dosage, and genetic risk score, *APOE* ε4 was associated with lower odds of fluctuating cognition and parkinsonism and higher odds of visual hallucinations. *APOE* ε4 was associated with greater neocortical Lewy body pathology, partially mediated by AD co‐pathology. The severity of fluctuating cognition was associated with Lewy body pathology stage after controlling for AD co‐pathology. Visual hallucinations were associated with both Lewy body and AD pathologies.

**DISCUSSION:**

Core clinical features of DLB are sensitive to *APOE* haplotype. Targeting *APOE* biology may elucidate DLB pathogenesis and inform therapeutic development.

**Highlights:**

- Core clinical features of dementia with Lewy bodies were sensitive to apolipoprotein E (*APOE*) haplotype.
- *APOE* ε4 was associated with a higher likelihood of hallucinations yet a lower likelihood of cognitive fluctuations.
- *APOE* ε4 was associated with greater severity of Lewy body pathology, partially mediated by Alzheimer's disease (AD) co‐pathology.
- Whereas fluctuating cognition was primarily linked to Lewy body pathology, visual hallucinations were associated with both Lewy body and AD neuropathologic changes.

## 1 BACKGROUND

Based on the current consensus criteria of the Dementia with Lewy Bodies Consortium, the clinical diagnosis of probable dementia with Lewy bodies (DLB) requires dementia and at least two of the four core clinical features, in the absence of indicative biomarkers: fluctuating cognition, visual hallucinations, parkinsonism, and rapid eye movement (REM) sleep behavior disorder (RBD).^1^ While genetic drivers of these core clinical features are not well understood, some genetic drivers would be anticipated to impact clinical expression through effects on Lewy body pathology, while others might do so through their effects on coincident Alzheimer's disease (AD) pathology, which accompanies DLB in an estimated 50% to 70% of cases.^2^, ^3^ While greater Lewy body pathology is associated with a worse profile of functional cognition in DLB,^4^ multiple studies have shown robust evidence that AD co‐pathology, when present, also contributes to greater impairment of functional cognition, as well as to a higher risk of mortality in DLB^5^ and a faster rate of cognitive decline in Lewy body disease (LBD).^6^

Apolipoprotein E (*APOE*) is a genetic factor that strongly affects AD risk, with *APOE* ε4 and *APOE* ε2 alleles, respectively, associated with increased and decreased risk of AD pathology.^7^ *APOE* ε4 has also been found to increase the risk of dementia in pure synucleinopathy.^8^ In DLB, *APOE* ε4 has been associated with cognitive impairment, including worse performance on tests of visuospatial function and executive function,^9^, ^10^ whereas *APOE* ε2 has been found to delay disease onset.^11^ In contrast to the well‐described association of *APOE* genotype and dementia in DLB, much less is known about whether *APOE* genotype impacts expression of DLB's core clinical features.

Previous studies have suggested that clinical detection of DLB may be inversely related to the presence of AD co‐pathology.^12^ Indeed, tau pathology in DLB has been linked to a lower frequency of parkinsonism and RBD.^13^ However, studies focusing on visual hallucinations have yielded conflicting results. While some studies have found a higher tau Braak stage to be associated with a lower risk of visual hallucinations in DLB,^14^, ^15^ others have not observed this association^13^ or have observed an increased likelihood of visual hallucinations in association with AD co‐pathology.^5^, ^16^ In addition, *APOE* ε4 in Parkinson's disease (PD) has been associated with increased odds of hallucinations.^17^ Although the basis for these heterogeneous observations is unclear, sample size has tended to be modest in these studies, and replication in large cohorts has been needed.

Despite its significant morbidity, fluctuating cognition in DLB has been much less explored than the other core clinical features of this disease. Fluctuating cognition is associated with impaired quality of life, drives greater disability and caregiver burden, and poses major challenges for assessing decision‐making capacity, highlighting the need to understand its underlying mechanisms and develop targeted therapies.^18^, ^19^ Although fluctuating cognition can also arise in AD, it tends to present very differently in these two diseases.^20^ In DLB, fluctuating cognition manifests as interruptions in awareness and attention that impair functional abilities as well as in wakefulness, while in AD, it typically manifests as transient confusion and communicative difficulties, typically triggered by environmental demands.^21^ In a recent study of DLB, the proportion of participants with fluctuating cognition trended lower in those with positive tau status.^13^ However, the association between *APOE* and fluctuating cognition in DLB has been unclear. Elucidating this association, along with *APOE*’s influence on other core clinical features, may facilitate the development of targeted symptomatic interventions that address individual features and ultimately improve outcomes for people with DLB.

**RESEARCH IN CONTEXT**

1. **Systematic review**: Apolipoprotein E (*APOE*) ε4 is well established as a major genetic risk factor for Alzheimer's disease (AD) neuropathologic changes. Its role in dementia with Lewy bodies (DLB), however, has been less thoroughly characterized. Previous studies investigating the effect of *APOE* on DLB have yielded mixed findings, particularly regarding associations with core clinical features and the interplay between AD co‐pathology and Lewy body pathology. As many DLB patients harbor concomitant AD‐related changes, a thorough understanding of *APOE* ε4's contribution to DLB has become critical.
2. **Interpretation**: *APOE* ε4 carriers exhibited lower odds of fluctuating cognition yet higher odds of visual hallucinations, and reduced odds of parkinsonism. *APOE* ε4 was associated with greater severity of Lewy body pathology, partially mediated by AD co‐pathology. While the severity of fluctuating cognition was linked primarily to Lewy body pathology, visual hallucinations were related to both Lewy body and AD pathologies.
3. **Future directions**: Future work should delineate the molecular pathways by which *APOE* ε4 modulates Lewy body and AD pathologies, determine its effects on brain circuits underlying Lewy body disease expression, and explore *APOE*‐targeted interventions. These findings may inform clinical trials and guide personalized therapeutic strategies for DLB.

Here, we examined the relationship between *APOE* genotype and the core clinical features of DLB across clinically probable DLB, AD, PD, and control groups, and we assessed the association of *APOE* genotype with Lewy body and AD neuropathological features in National Alzheimer Coordinating Center (NACC) autopsy cases. In addition to estimating the odds of these outcomes by *APOE* haplotype, we used a neuropathologically based *APOE* genetic risk score, recently developed as an indicator for AD pathology, that more accurately captures *APOE*’s effect in AD‐related analyses.^22^ We hypothesized that in DLB, *APOE* ε4 would be negatively associated with fluctuating cognition, parkinsonism, and RBD, but would be positively associated with visual hallucinations as reported in PD. We also hypothesized that *APOE* ε4 would contribute to Lewy body pathology in addition to AD co‐pathology.

## 2 METHODS

This was a cross‐sectional study using data collected in the large multicenter NACC Uniform Data Set (UDS) from September 2005 to October 2024.^23^ This large dataset included participant information collected from 46 past and present Alzheimer's Disease Research Centers (ADRCs) funded by the National Institute on Aging. All contributing ADRCs were required to obtain written informed consent from their participants and maintain their own separate evaluation and approval from their institutional review board before submitting data to the NACC.

### 2.1 Participants and measures

Participants were categorized into four groups based on previous clinical diagnosis: DLB, PD, AD, and control (CN) groups.^1^, ^24^, ^25^, ^26^ In the NACC dataset, diagnoses were assigned according to established clinical criteria: the consortium criteria for probable DLB,^1^, ^24^ the Movement Disorder Society (MDS) Task Force guidelines for PD,^26^ and the National Institute on Aging–Alzheimer's Association (NIA‐AA) workgroup guidelines for AD^25^ (https://naccdata.org/publish‐project/nacc‐references). The CN group comprised both healthy participants and those with other neurodegenerative disorders, given that participants with other neurodegenerative disorders were less likely to develop Lewy body symptoms or pathology. The diagnostic breakdown of the CN group is shown in Table S1 in supporting information. Participants were classified by their primary diagnosis at any study visit, as follows: (1) anyone with a primary DLB diagnosis at any visit, with or without AD as a contributing diagnosis, was classified as DLB; (2) of the remaining participants, anyone with a primary AD diagnosis at any visit, with or without DLB as a contributing diagnosis, was classified as AD; (3) PD and CN groups were defined using the same approach.

*APOE* genotype information was available for > 75% of participants in the UDS and was obtained from the National Centralized Repository for Alzheimer's Disease and Related Dementias. All participants with *APOE* data were classified as either *APOE* ε4 carriers or non‑carriers and, separately, as *APOE* ε2 carriers or non‑carriers. *APOE* ε4 dosage was defined as the number of *APOE* ε4 alleles present (0, 1, or 2). The *APOE* genetic risk score was a continuous variable derived from case–control analyses of participants carrying the *APOE* ε2/ε2, ε2/ε3, ε3/ε3, ε2/ε4, ε3/ε4, or ε4/ε4 genotype, and was a (naturally) logarithmically transformed measure of the odds ratio (OR) for AD pathology while adjusting for age and sex.^22^ *APOE* genetic risk score values for each genotype were as follows: ε2/ε2 = –1.833, ε2/ε3 = –0.916, ε3/ε3 = 0, ε2/ε4 = 0.904, ε3/ε4 = 1.742, and ε4/ε4 = 3.293.

The presence or absence of each core clinical feature was recorded for analysis as a binary variable. Fluctuating cognition was measured using the Mayo Fluctuation Scale (MFS), which ranges from 0 to 4. Participants scoring 3 or 4 were classified as having fluctuating cognition; those with lower scores were classified as not having fluctuating cognition.^21^ MFS is part of the NACC LBD module. Because the LBD module was administered only to participants suspected of or at risk for DLB or PD, data on fluctuating cognition were available for a subset of participants rather than the entire NACC cohort. Visual hallucinations were evaluated clinically as a binary variable indicating whether a participant exhibited a meaningful behavioral change attributed specifically to visual hallucinations. In the NACC dataset, the severity of hallucinations combined both visual and auditory components, preventing isolated assessment of the severity of visual hallucinations alone. Parkinsonism was recorded as a binary variable, indicating the presence or absence of parkinsonian signs. RBD was likewise assessed clinically as a binary variable reflecting the presence of dream enactment characteristic of this condition. Sample sizes differ across core clinical features because data were not available for all participants, and completeness varied across visits and forms. A clinical feature was considered present if it was reported at any time from the initial to the final visit in the NACC database. To investigate the association between fluctuating cognition severity and neuropathology, the MFS was taken from the visit closest to the time of autopsy. Symptom‐autopsy intervals were calculated from these timepoints.

The NACC neuropathology database includes ABC (Thal amyloid, Braak, Consortium to Establish a Registry for Alzheimer's Disease neuritic plaque) pathological scores for AD, as well as criteria for LBD, tauopathies, and frontotemporal lobar degeneration. Semiquantitative ratings were made by immunohistochemistry, histochemistry, microscopic visualization, or visual inspection and appropriate regional examinations. We classified AD neuropathologic change (ADNC) into four categories based on 2012 NIA‐AA criteria: not present(0), low (1), intermediate (2), and high (3).^27^ A participant with not present or low levels of ADNC was categorized as not having ADNC, whereas participants with intermediate or high levels were categorized as having ADNC. Lewy body pathology was stratified into four levels of staging: none (0), brainstem (1), limbic (2), and neocortical (3). We dichotomized participants into those having and those not having neocortical Lewy body pathology.

### 2.2 Study design and statistical analysis

This was a cross‐sectional study using data collected in the NACC UDS in which the primary dependent variables, usually tested in separate analyses, were (1) presence/absence of each of the four core clinical features of DLB: fluctuating cognition, visual hallucinations, parkinsonism, and RBD; and (2) severity of Lewy body pathology. The primary predictor terms were the four diagnostic groups (DLB, PD, AD, CN), *APOE* genotype measures (carriers, dosage, and genetic risk scores), and interactions between the diagnostic group and *APOE* measures. Diagnostic group and its interactions were first analyzed as 3 degree of freedom omnibus effects, followed up with simple effect tests and post hoc comparisons with adjustments of *P* values for multiple tests (e.g., with false discovery rate [FDR]).

Continuous dependent variables were analyzed with one‑way or factorial analysis of variance followed by post hoc pairwise tests. Bivariate relations among categorical variables were assessed using the chi‑squared test, or Fisher exact test when any expected cell count was below five. Logistic regression analyses were applied to assess the relation between *APOE* ε4 status (or dosage) and the presence of each of the four core clinical features, in all participants. Backward elimination logistic regression models were built with predictors of *APOE* ε4 status (or dosage), diagnostic group, and the interaction between *APOE* ε4 status (or dosage) and diagnostic group. For all analyses we conducted, if an interaction term was not significant, it was removed and the model rerun. Likelihood ratio tests were used to assess the significance of ORs in the multivariate analyses. In addition, we repeated these analyses with *APOE* status (or dosage) as a predictor in the DLB group only and in the PD group only. To further validate the findings from earlier steps, we built logistic regression models with *APOE* genetic risk score, diagnostic group, and the interaction between them, predicting the presence of each of the four core clinical features.

Ordinal logistic regression was applied to examine the relation between *APOE* genotype and Lewy body stage. The dependent variable was the severity of Lewy body pathology treated as an ordinal variable ranging in order of increasing severity from none to brainstem, limbic, and neocortical. The primary predictor terms were *APOE* ε4 status (or dosage), diagnostic group, and their interaction. Age, sex, and ADNC were included as covariates in the models. Because definitive diagnoses of DLB, PD, and AD require neuropathology as the gold standard, including clinical diagnostic groups in the model could introduce misclassification noise. Thus, we performed a second analysis in which we analyzed all cases with available neuropathologic and *APOE* data, regardless of clinical diagnosis. To determine whether the association between *APOE* ε4 and Lewy body pathology depends on AD pathology, we again built an ordinal logistic regression model with Lewy body severity as the dependent variable. The primary predictors were *APOE* ε4 status (or dosage), ADNC level, and their interaction. Age and sex were included as covariates.

We repeated these analyses in subgroups stratified by the ADNC levels if the test of the interaction term above indicated differential effects for them: one subgroup with ADNC, and another subgroup without ADNC. We also performed mediation analysis to examine the possible mediating effect of ADNC on the association between *APOE* genotype and Lewy body stage using R “mediation” package. The independent variable was *APOE* ε4 status, the mediator variable was the severity of ADNC, and the dependent variable was the presence of neocortical Lewy body stage. Age and sex were included as covariates.

Spearman correlation analyses were used to assess the relation between neuropathology and the severity of fluctuating cognition measured with the MFS. (Pearson correlations were not used because of the ordinal or binary nature of the variables involved.) Partial Spearman correlation was used to examine the effect of Lewy body pathology on the severity of fluctuating cognition after controlling for ADNC. Logistic regression analyses were applied to compare the frequency of Lewy body pathology, ADNC, and positive amyloid status measured by Pittsburgh compound B (PiB) positron emission tomography (PET), between those with and without fluctuating cognition. Logistic regression analyses were applied to compare the frequency of Lewy body pathology, and ADNC, between those with and without visual hallucinations.

All analyses in this study were conducted using R version 4.4.0. All tests were two tailed and were performed at a significance level of *P* < 0.05.

## 3 RESULTS

### 3.1 Participant characteristics

In total, 40,157 participants were included in this cross‐sectional study. Age, sex, and cognitive characteristics for each diagnostic group and sample sizes for assessment of each clinical feature are summarized in Table 1. The proportion of *APOE* ε4 carriers was ≈ 40% in DLB, 50% in AD, and 30% in PD and CN groups (Table S2 in supporting information). With respect to the core clinical features of DLB, 57.7% of DLB participants had fluctuating cognition, 56.3% experienced visual hallucination, and 65.9% had RBD. These proportions are higher than those observed in the AD, PD, and CN groups (each contrast, *P* < 0.001); 85.9% of DLB participants had motor manifestations of parkinsonism, a proportion lower than the 100% seen in PD but higher than observed in the AD and CN groups (*P* < 0.001).

**TABLE 1:** Demographics and characteristics of diagnostic groups.

	DLB	PD	AD	CN	*P* value
**Fluctuating cognition (N)**	163	42	39	101	
Age, mean (SD)	70.5 (7.5)	72.5 (8.3)	75.8 (10.4)	71.3 (10.2)	0.007^a^, ^b^
Female, *N* (%)	22 (13.5)	11 (26.2)	10 (25.6)	55 (54.5)	<0.001^a^, ^d^, ^f^
Presence, *N* (%)	94 (57.7)	9 (21.4)	2 (5.1)	4 (3.9)	<0.001^b^, ^d^, ^e^, ^f^
*APOE* ε4 carrier, *N* (%)	50 (30.7)	9 (21.4)	22 (56.4)	26 (25.7)	0.002^a^, ^b^, ^c^
*APOE*‐npscore, mean (SD)	0.5 (1.0)	0.2 (0.9)	1.1 (1.1)	0.4 (1.0)	0.001^a^, ^b^, ^c^
MoCA, *N*	157	37	36	98	
mean (SD)	20.7 (5.6)	24.6 (5.2)	19.0 (6.2)	26.3 (3.1)	<0.001^a^, ^c^, ^d^, ^e^
**Visual hallucinations (N)**	1208	1053	17957	19939	
Age, mean (SD)	72.0 (8.2)	72.6 (9.0)	73.7 (9.4)	68.4 (10.8)	<0.001^a^, ^b^, ^c^, ^d^, ^f^
Female, *N* (%)	294 (24.3)	342 (32.5)	9881 (55.0)	12186 (61.1)	<0.001^a^, ^b^, ^c^, ^d^, ^e^, ^f^
Presence, *N* (%)	680 (56.3)	261 (24.8)	1911 (10.6)	335 (1.7)	<0.001^a^, ^b^, ^c^, ^d^, ^e^, ^f^
*APOE* ε4 carrier, *N* (%)	516 (42.7)	313 (29.7)	9334 (52.0)	6193 (31.1)	<0.001^a^, ^b^, ^c^, ^d^, ^e^
*APOE*‐npscore, mean (SD)	0.7 (1.1)	0.4 (1.0)	1.0 (1.2)	0.5 (1.0)	<0.001^a^, ^b^, ^c^, ^d^, ^e^
MoCA, *N*	369	231	4681	7488	
Mean (SD)	20.3 (5.8)	23.9 (5.1)	19.0 (6.1)	25.1 (4.2)	<0.001^a^, ^b^, ^c^, ^d^, ^e^, ^f^
**Parkinsonism (N)**	534	469	8180	12911	
Age, mean (SD)	71.4 (8.2)	73.8 (8.8)	74.1 (9.7)	69.7 (10.6)	<0.001^a^, ^b^, ^d^, ^e^, ^f^
Female, *N* (%)	110 (20.6)	166 (35.4)	4375 (53.5)	7988 (61.9)	<0.001^a^, ^b^, ^c^, ^d^, ^e^, ^f^
Presence, *N* (%)	459 (85.9)	469 (100)	1258 (15.4)	1122 (8.7)	<0.001^a^, ^b^, ^c^, ^d^, ^e^, ^f^
*APOE* ε4 carrier, *N* (%)	217 (40.6)	127 (27.1)	4217 (51.6)	4064 (31.5)	<0.001^a^, ^b^, ^c^, ^d^, ^e^
*APOE*‐npscore, mean (SD)	0.6 (1.1)	0.4 (1.0)	1.0 (1.2)	0.5 (1.0)	<0.001^a^, ^b^, ^c^, ^d^, ^e^
MoCA, *N*	474	387	6765	11130	
mean (SD)	20.0 (6.0)	23.3 (5.6)	19.1 (6.3)	25.2 (4.2)	<0.001^a^, ^b^, ^c^, ^d^, ^e^, ^f^
**RBD (N)**	1032	843	15985	14953	
Age, mean (SD)	72.0 (8.2)	73.9 (9.0)	74.4 (9.8)	69.3 (10.8)	<0.001^a^, ^b^, ^d^, ^e^, ^f^
Female, *N* (%)	248 (24.0)	274 (32.5)	8717 (54.5)	9026 (60.4)	<0.001^a^, ^b^, ^c^, ^d^, ^e^, ^f^
Presence, *N* (%)	680 (65.9)	282 (33.5)	974 (6.1)	427 (2.9)	<0.001^a^, ^b^, ^c^, ^d^, ^e^, ^f^
*APOE* ε4 carrier, *N* (%)	441 (42.7)	251 (29.8)	8298 (51.9)	4666 (31.2)	<0.001^a^, ^b^, ^c^, ^d^, ^e^
*APOE*‐npscore, mean (SD)	0.7 (1.1)	0.4 (1.0)	1.0 (1.2)	0.5 (1.0)	<0.001^a^, ^b^, ^c^, ^d^, ^e^
MoCA	380	283	5171	10107	
mean (SD)	20.5 (5.8)	24.1 (5.0)	19.5 (6.1)	(3.9)	<0.001 ^a^, ^b^, ^c^, ^d^, ^e^ ^,f^

*Note*: Post hoc comparisons, *P* < 0.05. Age, age at first visit; MoCA, score at first visit in NACC. The number of participants with available data on fluctuating cognition was smaller than for other core features, reflecting that the MFS was administered only within the LBD module.

^a^ AD versus CN.

^b^ AD versus DLB.

^c^ AD versus PD.

^d^ DLB versus CN.

^e^ DLB versus PD.

^f^ PD versus CN.

Abbreviations: AD, Alzheimer's disease; ADNC, Alzheimer's disease neuropathologic change; *APOE*, apolipoprotein E; CN, control; DLB, dementia with Lewy bodies; MoCA, Montreal Cognitive Assessment; PD, Parkinson's disease; RBD, rapid eye movement behavior disorder; SD, standard deviation.

### 3.2 *APOE* ε4 carriers were less likely to exhibit fluctuating cognition and more likely to experience visual hallucinations

A higher *APOE* ε4 dosage was significantly associated with reduced odds of fluctuating cognition across all participants (OR: 0.49, 95% confidence interval [CI]: 0.28, 0.85, *P* = 0.009) and DLB participants alone (OR: 0.47, 95% CI: 0.25, 0.86, *P* = 0.015; Table 2). In addition, a higher *APOE* ε4 dosage was significantly associated with higher odds of visual hallucinations across all participants (OR: 1.30, 95% CI: 1.22, 1.37, *P* < 0.001), in DLB participants (OR: 1.26, 95% CI: 1.04, 1.52, *P* = 0.016), and in PD participants (OR: 1.35, 95% CI: 1.05, 1.72, *P* = 0.018). *APOE* ε4 dosage was also associated with reduced odds of parkinsonism across all participants (OR: 0.87, 95% CI: 0.81, 0.93, *P* < 0.001), and a trend toward reduced odds in DLB participants (OR: 0.68, 95% CI: 0.45, 1.02, *P* = 0.063). *APOE* ε4 dosage had no effect on the presence of RBD in DLB (OR: 0.85, 95% CI: 0.69, 1.04, *P* = 0.12) but was associated with significantly reduced odds in PD participants (OR: 0.67, 95% CI: 0.50, 0.89, *P* = 0.006).

**TABLE 2:** Effect of *APOE* ε4 dosage on the four core clinical features.

	All participants		Within DLB group only		Within PD group only	
*APOE* ε4 dosage	OR (95% CI)	*P* value	OR (95% CI)	*P* value	OR (95% CI)	*P* value
Fluctuating cognition	0.49 (0.28–0.85)	0.009^*^	0.47 (0.25–0.86)	0.015^*^	0.39 (0.04–3.62)	0.41
Visual hallucinations	1.30 (1.22–1.37)	<0.001^***^	1.26 (1.04–1.52)	0.016^*^	1.35 (1.05–1.72)	0.018^*^
Parkinsonism	0.87 (0.81‐0.93)	<0.001^***^	0.68 (0.45–1.02)	0.063	NA	NA
RBD	0.99 (0.93–1.08)^a^	0.99	0.85 (0.69–1.04)	0.12	0.67 (0.50–0.89)	0.006^**^

^a^ In all participants, the interaction between *APOE* ε4 dosage and diagnostic group is significant (*P* = 0.002), with PD group driving the effect of *APOE* ε4 dosage on the presence of RBD.

Abbreviations: *APOE*, apolipoprotein E; CI, confidence interval; DLB, dementia with Lewy bodies; OR, odds ratio; PD, Parkinson's disease; RBD, rapid eye movement behavior disorder.

Similar findings were observed comparing *APOE* ε4 carriers to non‐carriers (Table S3 in supporting information). In DLB participants, the odds of fluctuating cognition were reduced in those with *APOE* ε4 versus those without *APOE* ε4 (OR: 0.45, 95% CI: 0.23, 0.88, *P* = 0.020). In contrast, the odds of visual hallucinations in DLB tended toward an increase in those with *APOE* ε4 versus those without *APOE* ε4 (OR: 1.24, 95% CI: 0.98, 1.56, *P* = 0.069). The odds of parkinsonism tended to decrease in DLB with *APOE* ε4 versus those without *APOE* ε4 (OR: 0.66, 95% CI: 0.41, 1.08, *P* = 0.099). The odds of RBD in DLB did not differ between those with and without *APOE* ε4 (OR: 0.82, 95% CI: 0.63, 1.06, *P* = 0.12). Even so, in PD participants, the odds of developing RBD were reduced in those with *APOE* ε4 versus those without *APOE* ε4 (OR: 0.66, 95% CI: 0.47, 0.91, *P* = 0.011).

No significant association of *APOE* ε2 with clinical features was detected in the DLB or PD groups (Table S4 and Table S5 in supporting information). However, in all participants, *APOE* ε2 dosage was associated with reduced odds of visual hallucinations (OR: 0.80, 95% CI: 0.70, 0.91, *P* < 0.001), as was *APOE* ε2 status (OR: 0.79, 95% CI: 0.70, 0.91, *P* < 0.001). The interaction between *APOE* ε2 dosage (or status) and diagnostic group was significant (*P* < 0.001), with AD group driving the effect of *APOE* ε2 dosage (or status) on the presence of visual hallucination (*APOE* ε2 dosage OR: 0.64, 95% CI: 0.53, 0.76, *P* < 0.001; *APOE* ε2 carriers vs. non‐carriers OR: 0.63, 95% CI: 0.52, 0.76, *P* < 0.001).

### 3.3 *APOE* genetic risk score was linked to the presence of fluctuating cognition and visual hallucinations

**FIGURE 1:**
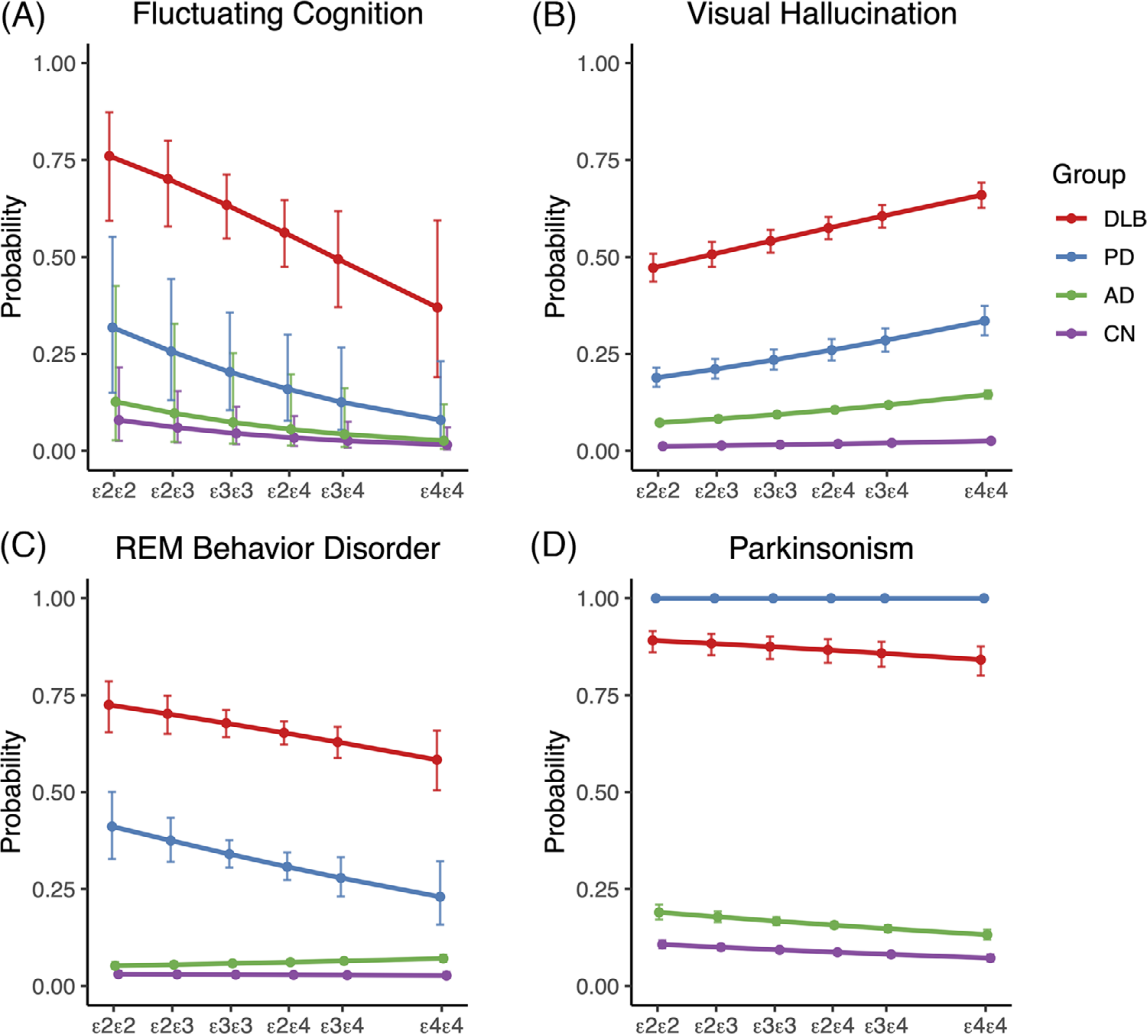
Association between *APOE* genetic risk score and core clinical features of DLB. A, As the *APOE* genetic risk score increased, the probability of developing fluctuating cognition decreased. B, Conversely, the odds of developing visual hallucinations increased with higher *APOE* genetic risk scores. C, For RBD, the odds decreased with increasing *APOE* genetic risk scores in DLB, while remaining consistently low in AD and CN groups. D, Increased *APOE* genetic risk score was associated with slightly reduced odds of parkinsonism. AD, Alzheimer's disease; *APOE*, apolipoprotein E; CN, control; DLB, dementia with Lewy bodies; PD, Parkinson's disease; RBD, rapid eye movement behavior disorder; REM, rapid eye movement

Across the diagnostic groups, the odds of developing fluctuating cognition decreased with higher *APOE* genetic risk score (OR: 0.69, 95% CI: 0.52, 0.93, *P* = 0.013; Figure 1A). Thus, fluctuating cognition was most common in participants harboring two copies of *APOE* ε2, and least common in participants harboring two copies of *APOE* ε4. In contrast, the odds of developing visual hallucinations across the diagnostic groups increased with higher *APOE* genetic risk score (OR: 1.16, 95% CI: 1.12, 1.20, *P* < 0.001), such that visual hallucinations were least common in participants harboring two copies of *APOE* ε2, and most common in participants harboring two copies of *APOE* ε4 (Figure 1B).

For RBD, the interaction term between group and *APOE* genetic risk score was significant (*P* = 0.004), indicating that the odds of RBD decreased with increased *APOE* genetic risk score in DLB (OR: 0.90, 95% CI: 0.81, 1.01, *P* = 0.079) and PD (OR: 0.86, 95% CI: 0.74, 0.99, *P* = 0.043), but remained low and stable in AD and CN (Figure 1C).

As the *APOE* genetic risk score increased, the odds of parkinsonism marginally decreased (OR: 0.93, 95% CI: 0.89, 0.96, *P* < 0.001, Figure 1D).

### 3.4 *APOE* ε4 was associated with Lewy body pathology, with partial dependence on AD pathology

We next evaluated the relationship between *APOE* haplotype and neuropathological findings. Odds of more severe Lewy body pathology were ≈ 17‐fold higher in DLB, 10‐fold higher in PD, and 1.5‐fold higher in AD relative to CN (all *P* < 0.001). Age showed no significant effect, whereas female sex was associated with 20% lower odds of advanced Lewy body pathology (OR: 0.80, 95% CI: 0.69, 0.93, *P* = 0.004). Compared to participants with no ADNC, greater Lewy body pathology was observed in participants with intermediate ADNC (OR: 1.89, 95% CI: 1.41, 2.53, *P* < 0.001) and high ADNC (OR: 2.10, 95% CI: 1.58, 2.80, *P* < 0.001). There was a significant interaction between *APOE* ε4 status and diagnostic group (*P* = 0.018), indicating that with CN as a reference group, AD *APOE* ε4 carriers tended toward increased Lewy body pathology compared to non‐carriers (OR: 1.55, 95% CI: 1.04, 2.31, *P* = 0.033; FDR‐adjusted *P* = 0.099), while associations between *APOE* ε4 and Lewy body pathology in DLB or PD were not significant. With PD as a reference group, AD *APOE* ε4 carriers tended to harbor increased Lewy body pathology compared to non‐carriers (OR: 2.38, 95% CI: 1.24, 4.57, *P* = 0.009; FDR‐adjusted *P* = 0.054). No such association in DLB was detected. Similar findings were observed with *APOE* ε4 dosage (data not shown). When we repeated the analysis restricting the control group to cognitively normal healthy controls only, the results remained similar (see Supplementary Material in supporting information).

**FIGURE 2:**
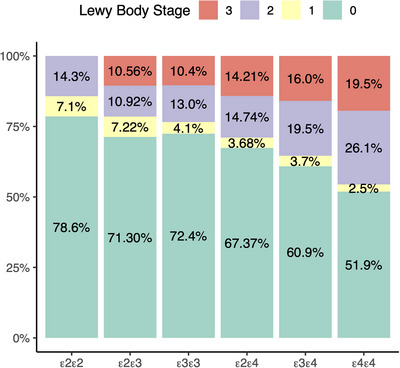
The effect of *APOE* ε4 on Lewy body pathology. *APOE* ε4 was associated with higher odds of advanced Lewy body pathology stages. This effect remained significant after adjusting for age, sex, and ADNC. Each additional *APOE* ε4 allele further elevated the odds of being in a higher Lewy body stage (*P* < 0.001). ADNC, Alzheimer's disease neuropathologic change; *APOE*, apolipoprotein E

Next, we set out to determine whether the association between *APOE* ε4 and Lewy body pathology was dependent on ADNC. The interaction between *APOE* ε4 and ADNC was not significant, and it was removed from the model. After adjusting for age, sex, and ADNC, *APOE* ε4 carriers had higher odds of more severe Lewy body pathology compared to non‐carriers (OR: 1.29, 95% CI: 1.11, 1.50, *P* < 0.001; Figure 2). For each additional increase in *APOE* ε4 dosage, the odds of being in a higher Lewy body stage increased after controlling for age, sex, and ADNC (OR: 1.26, 95% CI: 1.12, 1.42, *P* < 0.001).

Among those with intermediate to high levels of ADNC, *APOE* ε4 carriers had a significant association with more severe Lewy body pathology after adjusting for age and sex (OR: 1.26, 95% CI: 1.11, 1.43, *P* < 0.001), and constituted 60% of those with transitional and diffuse Lewy body pathology, compared to 51% of those with brainstem or no Lewy body pathology (Table 3). In contrast, among those with not present or low ADNC, *APOE* ε4 had a trending but non‐significant association with more severe Lewy body pathology (OR: 1.32, 95% CI: 0.97, 1.94, *P* = 0.073). *APOE* ε4 carriers comprised 23% of those with transitional and diffuse Lewy body pathology, compared to 18% in those with brainstem or no Lewy body pathology. In those with no ADNC, *APOE* ε4 was not associated with Lewy body pathology (OR: 1.50, 95% CI: 0.78, 2.87, *P* = 0.22).

**TABLE 3:** Characteristics of Lewy body and ADNC neuropathological groups.

	ADNC (*N* = 3712)			
	Not	Low	Intermediate	High
**No LBD**	460	579	573	1014
Age, years	70.1 (12.6)	73.1 (10.5)	79.2 (8.1)	72.4 (9.8)
Female, %	194 (42.2)	294 (50.8)	325 (56.7)	532 (52.5)
*APOE* ε4				
0	422 (91.7)	433 (74.8)	339 (59.2)	431 (42.5)
1	36 (7.8)	138 (23.8)	210 (36.6)	465 (45.9)
2	2 (0.5)	8 (1.4)	24 (4.2)	118 (11.6)
**Brainstem LBD**	29	63	55	33
Age, years	73.8 (9.4)	76.6 (9.5)	80.1 (7.9)	73.1 (10.1)
Female, %	14 (48.3)	23 (36.5)	25 (45.5)	12 (36.4)
*APOE* ε4				
0	26 (89.7)	49 (77.8)	31 (56.4)	18 (54.5)
1	3 (10.3)	14 (22.2)	22 (40.0)	14 (42.4)
2	0 (0)	0 (0)	2 (3.6)	1 (3.1)
**Transitional LBD**	36	45	70	213
Age, years	74.6 (11.9)	74.8 (8.8)	79.2 (7.5)	70.3 (9.5)
Female, %	15 (41.7)	16 (35.6)	35 (50.0)	108 (50.7)
*APOE* ε4				
0	34 (94.4)	30 (66.7)	46 (65.7)	79 (37.1)
1	2 (5.6)	14 (31.1)	22 (31.4)	98 (46.0)
2	0 (0)	1 (2.2)	2 (2.9)	36 (16.9)
**Diffuse LBD**	38	72	149	283
Age, years	71.9 (11.1)	72.4 (8.1)	74.8 (7.3)	72.4 (9.6)
Female, %	9 (23.7)	23 (31.9)	47 (31.5)	127 (44.9)
*APOE* ε4				
0	32 (84.2)	52 (72.2)	72 (48.3)	91 (32.2)
1	5 (13.2)	19 (26.4)	61 (40.9)	147 (51.9)
2	1 (2.6)	1 (1.4)	16 (10.8)	45 (15.9)

Abbreviations: ADNC, Alzheimer's disease neuropathologic change; *APOE*, apolipoprotein E; LBD, Lewy body disease.

Mediation analysis revealed that ADNC partially mediated the relationship between *APOE* and Lewy body stage. The total effect of *APOE* ε4 on harboring neocortical Lewy body pathology was significant (β = 0.34, *P* < 0.001). After including ADNC in the model, the direct effect of *APOE* ε4 on neocortical Lewy body pathology remained significant (β = 0.21, *P* < 0.001), while the indirect effect through ADNC was also significant (β = 0.13, *P* < 0.001). Approximately 39% of the total effect was mediated by ADNC, indicating partial mediation. This observation suggests that while ADNC explains part of the effect of *APOE* ε4 on neocortical Lewy body pathology, other factors also contribute.

### 3.5 Lewy body pathology, but not AD pathology, was associated with fluctuating cognition

**FIGURE 3:**
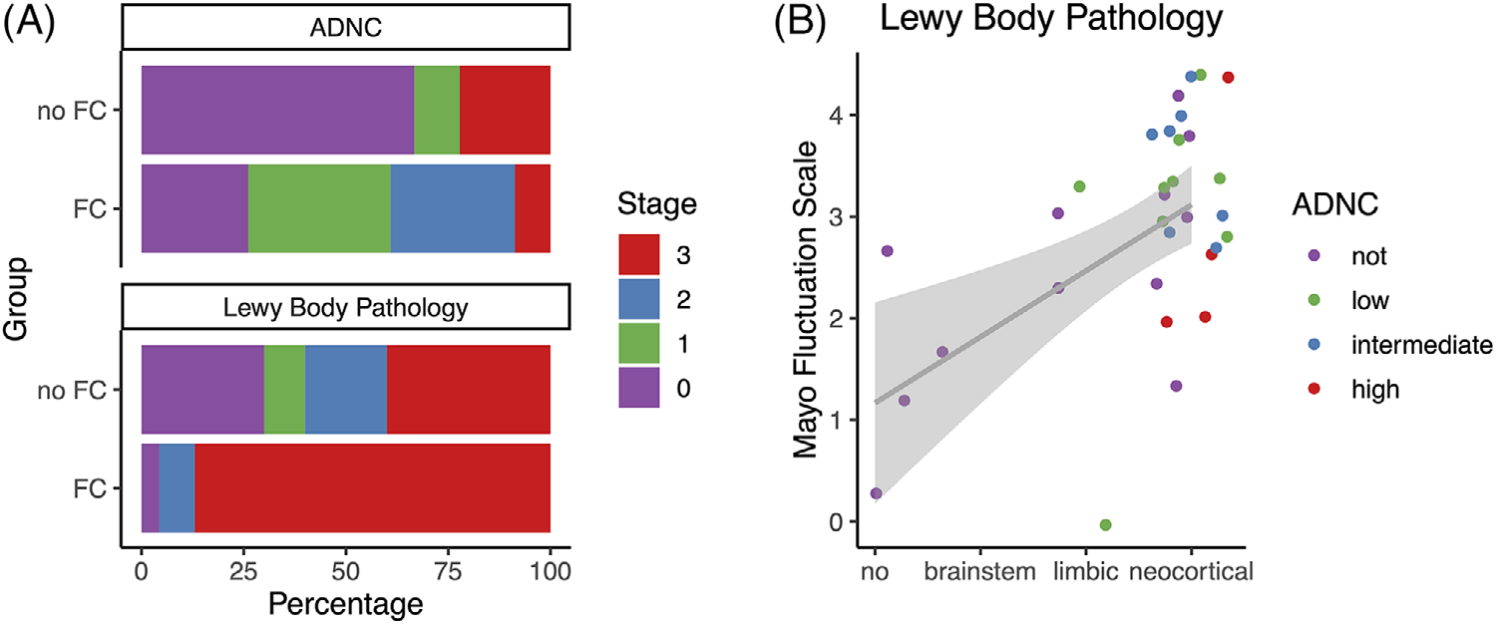
The association of fluctuating cognition with neuropathological changes. A, Fluctuating cognition (FC) was positively correlated with the severity of Lewy body pathology but not with ADNC. Participants with fluctuating cognition were more likely to exhibit a neocortical Lewy body stage, while the proportion of ADNC was similar between those with and without fluctuating cognition. B, Scatterplot of Mayo Fluctuations Scale score versus Lewy body pathology. ADNC, Alzheimer's disease neuropathologic change; FC, fluctuating cognition

The proportion of participants harboring neocortical Lewy body pathology was higher in participants with fluctuating cognition than in those without (*χ^2^ *= 8.9, *P* = 0.031). Participants with fluctuating cognition more frequently harbored neocortical stage Lewy body pathology than those without fluctuating cognition (OR: 10.0, 95% CI: 1.7, 57.7, *P* = 0.007; Figure 3A). In contrast, the odds of harboring ADNC were similar across those with and without fluctuating cognition (*χ^2^ *= 0.24, *P* = 0.62; Figure 3A). Similarly, no difference in amyloid status measured with PiB PET was observed between those with and without fluctuating cognition (*χ^2 ^* = 0.13, *P* = 0.71). Conversely, the proportion of participants presenting with fluctuating cognition did not vary with amyloid status (*χ^2^ *= 0.17, *P* = 0.68; *N* = 144).

The severity of fluctuating cognition measured by the MFS at last visit moderately correlated with the severity of Lewy body pathology (Spearman rho = 0.55, *P* < 0.001; Figure 3B), and this result persisted after adjusting for symptom–autopsy interval (rho = 0.54, *P* = 0.001). In contrast, the severity of fluctuating cognition was not associated with the severity of ADNC (rho = 0.31, *P* = 0.09). The association of Lewy body pathology with the MFS remained significant after controlling for ADNC (rho = 0.45, *P* = 0.012).

In the subgroup of clinically diagnosed DLB participants who underwent autopsy, despite a limited sample size (*N* = 23), the severity of fluctuating cognition was still significantly associated with Lewy body pathology (rho = 0.43, *P* = 0.036), and this result persisted after adjusting for symptom–autopsy interval (rho = 0.43, *P* = 0.041). In DLB cases, as observed overall, there was no association of MFS with the severity of ADNC (rho = 0.13, *P* = 0.54). The association of Lewy body pathology with the MFS in DLB did not survive after controlling for ADNC (rho = 0.34, *P* = 0.12).

### 3.6 Both Lewy body and AD pathologies contributed to the presence of visual hallucinations

The proportion of participants harboring neocortical Lewy body pathology was higher in participants with visual hallucinations than in those without (*χ^2^ *= 238.4, *P* < 0.001). Similarly, ADNC was more severe in those with visual hallucinations compared to those without (*χ^2^ *= 114.4, *P* < 0.0001). Conversely, compared to participants without visual hallucinations, participants experiencing visual hallucinations demonstrated a higher prevalence of neocortical Lewy body disease (OR: 4.6, 95% CI: 3.8, 5.5, *P* < 0.0001), as well as intermediate to high ADNC (OR: 2.3, 95% CI: 1.9, 2.8, *P* < 0.0001).

Similar results were observed among DLB and AD participants. Those with visual hallucinations exhibited more severe Lewy body pathology (DLB: *χ^2^ *= 19.8, *P* = 0.0002; AD: *χ^2^ *= 57.6, *P* < 0.0001) and more severe AD pathology (DLB: *χ^2^ *= 3.2, *P* = 0.074; AD: *χ^2^ *= 63.7, *P* < 0.0001) compared to those without.

## 4 DISCUSSION

Informed by the effect of *APOE* ε4 on AD risk and the frequent presence of AD co‐pathology in DLB, we used the NACC UDS to investigate the associations between *APOE* ε4 and core clinical features and neuropathology in DLB. Our findings demonstrate that *APOE* ε4 is associated with a lower likelihood of fluctuating cognition and parkinsonism, yet a higher likelihood of visual hallucinations. These observations were consistent in analyses of *APOE* ε4 carriers, *APOE* ε4 dosage, and *APOE* genetic risk scores. The large size of this study sample provided sufficient statistical power to estimate the ORs for each core clinical feature of DLB in relation to *APOE* ε4 status. This study also provides further evidence that Lewy body pathology, but not AD pathology, is associated with the presence and the severity of fluctuating cognition, while both Lewy body and AD pathologies contribute to the presence of visual hallucinations. Interestingly, the association between *APOE* genotype and Lewy body pathology was found to be partially mediated by AD pathology, expanding the therapeutic potential of targeting *APOE* for DLB. Together, these findings highlight the value for incorporating *APOE* genotype into clinical trial stratification and treatment evaluation. Further, they suggest that therapeutic targeting of α‐synuclein pathology may have benefit for fluctuating cognition, while combination therapy targeting α‐synuclein and AD pathologies may be most beneficial against hallucinations.

In this cohort, fluctuating cognition was common in DLB, affecting two thirds of DLB participants, a higher proportion than observed in PD, AD, and CN. As fluctuating cognition has been linked to greater impairment in daily activities and to a diminished quality of life,^28^ understanding the biological mechanisms of fluctuating cognition is of critical importance. Our finding that *APOE* ε4 is associated with a lower likelihood of fluctuating cognition may prove useful in this regard. We also observed a significant association between fluctuating cognition and Lewy body pathology, even after controlling for AD pathology. This finding, which focuses on the variation in alertness and attention that arises in DLB and is reflected in the MFS score,^20^ aligns with and complements a prior report demonstrating that neocortical Lewy pathology is associated with variability in the trajectory of cognitive decline as a distinct measure of fluctuating cognition, independent of AD pathology.^29^

Our observation that *APOE* ε4 is associated with higher odds of visual hallucinations, arising from both Lewy body and AD pathologies, is consistent with the frequent presence of visual hallucinations in Lewy body disease, as well as with their capacity to manifest in the later stages of AD.^30^, ^31^ Interestingly, participants with dual AD and LBD pathology have been reported to have the highest risk of visual hallucinations.^16^, ^32^ Previous reports have linked the severity of fluctuating cognition and the presence of visual hallucinations to the integrity of the nucleus basalis of Meynert,^33^, ^34^ building from the hypothesis that cholinergic degeneration, a consistent and substantial pathological finding in Lewy body disease, may contribute. Moreover, *APOE* ε4 has been implicated in impairing synaptic integrity and promoting cholinergic dysfunction,^35^, ^36^ processes crucial for visual processing that when impaired are thought to contribute to visual hallucinations.^37^

Our observations that *APOE* ε4 was associated with Lewy body pathology, and that this relationship was partially mediated by AD pathology, is supported by reports that *APOE* ε4 exacerbates α‐synuclein pathology independently of amyloid pathology^38^ and directly modulates its progression, correlating with accelerated cognitive decline.^39^  Although *APOE* ε4 has been consistently associated with the presence of AD pathology, the presence of an *APOE* ε4 allele in AD has also been associated with the presence of concomitant Lewy bodies,^40^ and *APOE* ε4 has been found to exacerbate α‐synuclein seeding activity and neurotoxicity.^41^ In DLB, the *APOE* ε4 allele is also associated with greater neuritic degeneration in the CA2 and CA3 subfields, where Lewy bodies are frequently detected.^42^, ^43^ Even so, the impact of *APOE* ε4 on α‐synuclein pathology in pure LBD remains controversial.^44^, ^45^, ^46^, ^47^

The strengths of this study include the use of the large, well‐characterized NACC cohort; integration of genetic, clinical, and neuropathological data; and the use of *APOE* ε4 carriers, *APOE* dosage, as well as *APOE* genetic risk scores to validate results. This study also has several limitations. First, diagnostic groups were based on clinical diagnosis. Accuracy of a clinical diagnosis of probable DLB, AD, and PD is acceptable, and several analyses were performed in autopsy‐proven cases.^48^, ^49^, ^50^ In this dataset, 82% of clinically diagnosed DLB and PD had Lewy body pathology, and 78% of clinically diagnosed AD had intermediate‐to‐high AD neuropathologic change. Second, the sample size for analyses of fluctuating cognition was relatively small compared to other clinical features. Although this limitation reduces statistical power, we were still able to detect a significant association between fluctuating cognition and *APOE* genotypes. However, because the MFS was administered only to a subset of participants that received the NACC LBD module, the sample may have been biased toward individuals suspected of or at risk for DLB or PD. Thus, further studies with systematically collected data across diagnostic groups are needed to validate the observed association between *APOE* ɛ4 and a lower likelihood of fluctuating cognition. Third, the *APOE* genetic risk score was derived from a different, reference population. Even so, this metric has been shown to be a robust index, superior to *APOE* ε4 counts, for indicating AD pathology,^22^ and the demographic characteristics of that reference population were broadly comparable to our cohort. Fourth, disease duration and medication use may influence the presence of the core clinical features of DLB assessed here. To contend with this issue, we defined the presence of each symptom for a given participant as any report of the symptom across all visits. As this approach partially mitigates but does not entirely eliminate possible drug effects, our findings warrant further validation from future studies with longitudinal design. Another limitation is that the NACC cohort includes relatively few PD participants, as it was not primarily designed to study this population; validation in larger cohorts with substantial PD representation will be of value. Finally, the cohort was predominantly non‐Hispanic White (Table S6 in supporting information), which may limit the generalizability of our findings to more diverse populations.

In conclusion, the results of this study show that the core clinical features of DLB are sensitive to *APOE* haplotype, with *APOE* ε4 associated with a higher likelihood of hallucinations yet a lower likelihood of cognitive fluctuations. Whereas fluctuating cognition is primarily linked to Lewy body pathology, visual hallucinations appear to be driven by both Lewy body and AD pathology. Elucidating the mechanisms underlying these effects of *APOE* haplotype may offer new insights into disease pathobiology and set the stage for more effective treatment strategies for this recalcitrant disease.

## CONFLICT OF INTEREST STATEMENT

R.Y., A.E.G., J.J.L., E.C.P., P.S., Y.W., and S.N.G. report no conflicts of interest relevant to the present study. Author disclosures are available in the supporting information.

## CONSENT STATEMENT

The NACC protocols were approved by each cohort's respective institutional ethical review board. All participants provided written informed consent. The studies were performed in accordance with the Declaration of Helsinki and its later amendments.

## Supporting information



Supporting Information

Supporting Information
