# Supplementary material for: APOE ε4 linked effects on clinical features and neuropathology in dementia with Lewy bodies
Source: Alzheimers Dement. 2025 Oct 11;21(10):e70795. doi: 10.1002/alz.70795 (PMC12514938; doi:10.1002/alz.70795)
Supplement: Supplementary file 1 — Supporting Information [file ALZ-21-e70795-s001.docx]

**Supplementary Material**

**Results**

**APOE ε4 was associated with Lewy body pathology, with partial dependence on AD pathology**

Odds of more severe Lewy body pathology were about 13-fold higher in DLB (p < 0.001), 8-fold higher in PD (p < 0.001), and 1.3-fold higher in AD (p = 0.088) relative to cognitively normal healthy controls. Age showed no significant effect, whereas female sex was associated with 22% lower odds of advanced Lewy body pathology (OR: 0.78, 95%CI: 0.66, 0.91, p = 0.002). Compared to participants with not ADNC, greater Lewy body pathology was observed in participants with intermediate ADNC (OR: 1.66, 95%CI: 1.21, 2.31, p = 0.002) and high ADNC (OR: 1.67, 95%CI: 1.21, 2.30, p = 0.002). There was a significant interaction between APOE ε4 status and diagnostic group (p = 0.008), indicating that with cognitively normal healthy controls as a reference group, AD APOE ε4 carriers had increased Lewy body pathology compared to non-carriers (OR: 2.15, 95%CI: 1.17, 3.93, p = 0.014; FDR adjusted p = 0.042), while associations between APOE ε4 and Lewy body pathology in DLB or PD were not significant. With PD as a reference group, AD APOE ε4 carriers had increased Lewy body pathology compared to non-carriers (OR: 2.33 95%CI: 1.22, 4.45, p = 0.011; FDR adjusted p = 0.042). No such association in DLB was detected.

**Supplementary Table 1. Diagnostic breakdown of CN group**

| Fluctuating cognition | | Visual hallucination | | Parkinsonism | | RBD | |
| --- | --- | --- | --- | --- | --- | --- | --- |
| Primary Dx | N, % | Primary Dx | N, % | Primary Dx | N, % | Primary Dx | N, % |
| HC | 84 (83) | HC | 13901 (70) | HC | 9836 (76) | HC | 9800 (66) |
| FTLD | 2 (2) | FTLD | 1836 (9) | FTLD | 805 (6) | FTLD | 1660 (11) |
| PSP | 1 (1) | PSP | 203 (1) | PSP | 104 (1) | PSP | 189 (1) |
| CBD | 2 (2) | CBD | 239 (1) | CBD | 114 (1) | CBD | 202 (1) |
| MSA | 0 (0) | MSA | 1 (0) | MSA | 1 (0) | MSA | 1 (0) |
| Other | 12 (12) | Other | 3759 (19) | Other | 2051 (16) | Other | 3101 (21) |

HC, cognitively normal healthy control; FTLD, Frontotemporal lobar degeneration; PSP, Progressive supranuclear palsy; CBD, Corticobasal degeneration; MSA, Multiple system atrophy

**Supplementary Table 2. APOE genotype frequencies by clinical group, for each symptom**

| Clinical group | Participant, N | APOE genotype | | | | | |
| --- | --- | --- | --- | --- | --- | --- | --- |
|  |  | 2/2 | 2/3 | 3/3 | 2/4 | 3/4 | 4/4 |
| **Fluctuating Cognition, N (%)** | **345** | **3 (1)** | **29 (8)** | **206 (60)** | **8 (2)** | **88 (26)** | **11 (3)** |
| DLB | 163 | 1 (1) | 14 (9) | 98 (60) | 3 (2) | 43 (26) | 4 (2) |
| PD | 42 | 1 (2) | 4 (10) | 28 (67) | 1 (2) | 8 (19) | 0 (0) |
| AD | 39 | 0 (0) | 1 (3) | 16 (41) | 2 (5) | 16 (41) | 4 (10) |
| CN | 101 | 1 (1) | 10 (10) | 64 (63) | 2 (2) | 21 (21) | 3 (3) |
| **Visual Hallucinations, N (%)** | **40157** | **174 (0)** | **3577 (9)** | **20050 (50)** | **1034 (3)** | **12625 (31)** | **2697 (7)** |
| DLB | 1208 | 2 (0) | 94 (8) | 596 (49) | 51 (4) | 387 (32) | 78 (6) |
| PD | 1053 | 9 (1) | 117 (11) | 614 (58) | 23 (2) | 252 (24) | 38 (4) |
| AD | 17957 | 45 (0) | 1136 (6) | 7442 (41) | 485 (3) | 6922 (39) | 1927 (11) |
| CN | 19939 | 118 (1) | 2230 (11) | 11398 (57) | 475 (2) | 5064 (25) | 654 (3) |
| **Parkinsonism, N (%)** | **22094** | **95 (1)** | **2075 (9)** | **11299 (51)** | **551 (3)** | **6718 (30)** | **1356 (6)** |
| DLB | 534 | 2 (0) | 51 (10) | 264 (49) | 25 (5) | 171 (32) | 21 (4) |
| PD | 469 | 2 (0) | 48 (10) | 292 (62) | 8 (2) | 107 (23) | 12 (3) |
| AD | 8180 | 20 (1) | 524 (6) | 3419 (42) | 199 (2) | 3136 (38) | 882 (11) |
| CN | 12911 | 71 (1) | 1452 (11) | 7324 (57) | 319 (2) | 3304 (26) | 441 (3) |
| **RBD, N (%)** | **32813** | **131 (0)** | **2859 (9)** | **16167 (49)** | **845 (3)** | **10487 (32)** | **2324 (7)** |
| DLB | 1032 | 2 (0) | 82 (8) | 507 (49) | 46 (4) | 325 (31) | 70 (7) |
| PD | 843 | 7 (1) | 85 (10) | 500 (59) | 22 (3) | 201 (24) | 28 (3) |
| AD | 15985 | 40 (0) | 1016 (6) | 6631 (41) | 427 (3) | 6147 (38) | 1724 (11) |
| CN | 14953 | 82 (1) | 1676 (11) | 8529 (57) | 350 (2) | 3814 (26) | 502 (3) |

**Supplementary Table 3. Effect of APOE ε4 carriers vs. non-carriers** **on the four core clinical features**

| APOE ε4 carriers vs. non-carriers | All participants | | Within DLB group only | | Within PD group only | |
| --- | --- | --- | --- | --- | --- | --- |
|  | OR (95% CI) | P value | OR (95% CI) | P value | OR (95% CI) | P value |
| Fluctuating cognition | 0.43 (0.23-0.80) | 0.006^*^ | 0.45 (0.23-0.88) | 0.020^*^ | 0.39 (0.04-3.62) | 0.41 |
| Visual hallucination | 1.36 (1.28-1.47) | <0.001^***^ | 1.24 (0.98-1.56) | 0.069 | 1.28 (0.95-1.73) | 0.11 |
| Parkinsonism | 0.85 (0.78-0.93) | <0.001^***^ | 0.66 (0.41-1.08) | 0.099 | NA | NA |
| RBD | 1.01 (0.92-1.11)^#^ | 0.82 | 0.82 (0.63-1.06) | 0.12 | 0.66 (0.47-0.91) | 0.011^*^ |

#, In all participants, the interaction between APOE ε4 status and diagnostic group is significant (p = 0.002), with PD group driving the effect of APOE ε4 carriers on the presence of RBD.

**Supplementary Table 4. Effect of APOE ε2** **dosage** **on the four core clinical features**

| APOE ε2 dosage | All participants | | Within DLB group only | | Within PD group only | |
| --- | --- | --- | --- | --- | --- | --- |
|  | OR (95% CI) | P value | OR (95% CI) | P value | OR (95% CI) | P value |
| Fluctuating cognition | 1.13 (0.53-2.43) | 0.75 | 1.01 (0.40-2.52) | 0.98 | 2.54 (0.57-11.3) | 0.22 |
| Visual hallucination | 0.80 (0.70-0.91)^#^ | <0.001^***^ | 0.81 (0.58-1.14) | 0.23 | 1.22 (0.86-1.73) | 0.27 |
| Parkinsonism | 1.06 (0.94-1.19) | 0.35 | 1.53 (0.71-3.26) | 0.28 | NA | NA |
| RBD | 0.87 (0.76-1.01) | 0.071 | 1.26 (0.85-1.87) | 0.25 | 0.74 (0.49-1.11) | 0.15 |

#, In all participants, the interaction between APOE ε2 dosage and diagnostic group is significant (p < 0.001), with AD group driving the effect of APOE ε2 dosage on the presence of visual hallucination.

**Supplementary Table 5. Effect of APOE ε2** **carriers vs. non-carriers** **on the four core clinical features**

| APOE ε2 carriers vs. non-carriers | All participants | | Within DLB group only | | Within PD group only | |
| --- | --- | --- | --- | --- | --- | --- |
|  | OR (95% CI) | P value | OR (95% CI) | P value | OR (95% CI) | P value |
| Fluctuating cognition | 1.13 (0.49-2.64) | 0.77 | 0.91 (0.34-2.43) | 0.85 | 5.0 (0.81-31) | 0.084 |
| Visual hallucination | 0.79 (0.70-0.91)^#^ | <0.001^***^ | 0.84 (0.59-1.18) | 0.31 | 1.28 (0.87-1.88) | 0.22 |
| Parkinsonism | 1.06 (0.93-1.20) | 0.39 | 1.51 (0.69-3.27) | 0.30 | NA | NA |
| RBD | 0.89 (0.76-1.03) | 0.12 | 1.24 (0.83-1.85) | 0.29 | 0.75 (0.48-1.16) | 0.19 |

#, In all participants, the interaction between APOE ε2 status and diagnostic group is significant (p < 0.001), with AD group driving the effect of APOE ε2 carriers on the presence of visual hallucination.

**Supplementary Table 6. Distribution of race and Hispanic ethnicity across diagnostic groups**

|  | DLB | PD | AD | CN | P value |
| --- | --- | --- | --- | --- | --- |
| **Fluctuating Cognition (N)** | 163 | 42 | 39 | 101 |  |
| Race/Ethnicity, N (%) |  |  |  |  | <0.001 |
| Non-Hispanic White | 155 (95.1) | 39 (92.8) | 31 (79.5) | 82 (81.1) |  |
| Non-Hispanic Black | 4 (2.5) | 1 (2.4) | 1 (2.6) | 1 (1.0) |  |
| Hispanic | 1 (0.6) | 2 (4.8) | 2 (5.1) | 9 (8.9) |  |
| Asian | 1 (0.6) | 0 (0) | 4 (10.2) | 5 (5.0) |  |
| Other | 2 (1.2) | 0 (0) | 1 (2.6) | 4 (4.0) |  |
| **Visual Hallucinations (N)** | 1208 | 1053 | 17957 | 19939 |  |
| Race/Ethnicity, N (%) |  |  |  |  | <0.001 |
| Non-Hispanic White | 1038 (85.9) | 913 (86.7) | 13821 (77.0) | 14264 (71.6) |  |
| Non-Hispanic Black | 62 (5.1) | 34 (3.2) | 1840 (10.2) | 2778 (13.9) |  |
| Hispanic | 60 (5.0) | 53 (5.0) | 1422 (7.9) | 1605 (8.0) |  |
| Asian | 16 (1.3) | 27 (2.6) | 404 (2.3) | 583 (2.9) |  |
| Other | 32 (2.7) | 26 (2.5) | 470 (2.6) | 709 (3.6) |  |
| **Parkinsonism (N)** | 534 | 469 | 8180 | 12911 |  |
| Race/Ethnicity, N (%) |  |  |  |  | <0.001 |
| Non-Hispanic White | 471 (88.2) | 395 (84.2) | 6278 (76.7) | 9017 (69.8) |  |
| Non-Hispanic Black | 23 (4.3) | 12 (2.6) | 800 (9.8) | 1908 (14.8) |  |
| Hispanic | 24 (4.5) | 33 (7.0) | 662 (8.1) | 1110 (8.6) |  |
| Asian | 7 (1.3) | 17 (3.6) | 229 (2.8) | 425 (3.3) |  |
| Other | 9 (1.7) | 12 (2.6) | 211 (2.6) | 451 (3.5) |  |
| **RBD (N)** | 1032 | 843 | 15985 | 14953 |  |
| Race/Ethnicity, N (%) |  |  |  |  | <0.001 |
| Non-Hispanic White | 889 (86.1) | 720 (85.4) | 12299 (76.9) | 10632 (71.1) |  |
| Non-Hispanic Black | 53 (5.1) | 31 (3.7) | 1601 (10.0) | 2064 (13.8) |  |
| Hispanic | 49 (4.8) | 47 (5.6) | 1288 (8.1) | 1251 (8.4) |  |
| Asian | 15 (1.5) | 22 (2.6) | 374 (2.3) | 470 (3.1) |  |
| Other | 26 (2.5) | 23 (2.7) | 423 (2.6) | 536 (3.6) |  |
